# Transcroporal Artificial Urinary Sphincter Placement With Closure of Corporal Bodies—A Long-Term Analysis of Functional Outcomes

**DOI:** 10.3389/fsurg.2022.918011

**Published:** 2022-06-01

**Authors:** Valentin Maurer, Roland Dahlem, Marian Howaldt, Silke Riechardt, Margit Fisch, Tim A. Ludwig, Oliver Engel

**Affiliations:** ^1^ Department of Urology, University Medical Center Hamburg-Eppendorf, Hamburg, Germany; ^2^ Department of Urology, Asklepios Medical Center Hamburg-Harburg, Hamburg, Germany

**Keywords:** AMS 800, artificial urinary sphincter, reconstructive urology, stress urinary, transcorporal cuffs, corporal closure

## Abstract

**Objectives:**

An artificial urinary sphincter (AUS) is the gold standard for postoperative stress urinary incontinence (SUI). The transcorporal AUS (TC) placement constitutes the main salvage option in high-risk patients suffering from SUI with fragile urethras. The literature analyzing long-term outcomes with respect to explantation rates, continence, and erectile function is scarce.

**Methods and Patients:**

Retrospective data collection was performed in 2011. TC was applied according to a standardized protocol. TC was implanted after bulbar urethroplasty or double-cuff (DC) explantation. After TC placement, the tunica albuginea was closed in order to minimize the risk of postoperative bleedings and erectile dysfunction. Activation was performed 6 weeks postoperatively. Further follow-up (FU) was scheduled 6/24 months postoperatively and every 2 years thereafter. Primary/secondary endpoints were explantation/objective, subjective, and social continence rates. Objective or social continence was defined as the use of 0 pads/day or <2 pads/day, respectively. Thereupon, postoperative bleedings and erectile function were analyzed.

**Results:**

A total of 39 high-risk patients were available for analysis. The median age was 72 years. In total, 84.6%, 10.3%, and 2.6% had a history of radical prostatectomy, TURP, and radical cystectomy, respectively. In total, 61.5% had a history of radiation therapy of the prostate, 41% had a history of urethral surgery, and 95% had a history of double cuff explantation. The median FU was 27 months. Objective, subjective, and social continence were 54.5%, 69.7%, and 78.8%, respectively. The median pad usage was 1 pad/day [1–2.5]. Only one patient suffered from a postoperative hematoma. In total, 15.4% of the patients were able to have an erection preoperatively, compared to 7.7% after TC placement. The estimated mean explantation-free survival of the TC was 83 months in the Kaplan–Meier analysis.

**Conclusions:**

TC AUS implantation constitutes a viable salvage approach in high-risk SUI patients with a mean device survival of almost 7 years and high social continence rates of almost 80%. An intraoperative closure of the tunica albuginea after TC placement allows for very low rates of postoperative hematoma and supports postoperative erectile rigidity.

## Introduction

Male stress urinary incontinence (SUI) is a feared complication after local surgical treatment or irradiation of prostate cancer and after surgical treatment of benign prostatic hyperplasia. Incontinence rates range from about 20% to up to 36% one year after radical prostatectomy (RP) (1, 2).

Artificial urinary sphincter (AUS) implantation constitutes the gold standard in the surgical treatment of SUI (3, 4).

According to the current body of the literature, surgical sequencing in accordance with the patients’ medical history—starting with a membranous SC, followed by a distal double cuff (DC) with a TC as the ultimate salvage option—constitutes the state-of-the-art approach to the treatment of severe SUI (5, 6).

AUS implantation in general is associated with adverse events, such as erosion, mechanical failure, and infection, all of which lead to revision surgery or to the explantation of the device (7, 8). TC-AUS implantation as a salvage option in patients with a fragile urethra is in particular associated with a loss of erectile rigidity (9) and a higher risk of postoperative hematoma due to the dissection of the corpora cavernosa. However, it has been discussed in the literature that a closure of the tunica albuginea may reduce the incidence of the latter complications (10).

This brings up the question of whether there is a particular superior surgical technique that serves the surgeons as a viable salvage option with respect to continence, explantation rates, and erectile function.

To our knowledge, this study is the first to assess the hypothesis that the closure of the tunica albuginea reduces the risk of postoperative bleedings and improves postoperative erectile rigidity even in high-risk cases.

## Patients and Methods

### Patient Population

Since January 2011, in accordance with an Institutional Review Board approval, all perioperative and follow-up (FU) data of patients undergoing AUS implantation (AMS 800) at our institution have been collected in an AUS database. We included male patients with severe SUI according to the international continence society (11). Patients with detrusor overactivity or insufficient compliance apparent during the first 300 ml of bladder filling at preoperative urodynamic cystomanometry or those with mild SUI (i.e., male sling patients) were excluded prior to analyses. Moreover, patients suffering from insufficient manual dexterity (based on a standardized ballpoint pen dis- and reassembling test at our institution) in the preoperative evaluation were excluded.

A TC approach is applied if patients have a history of a DC explantation or if patients have a history of bulbar urethroplasty. The time gap to the last urethral manipulation (i.e., AUS explantation or bulbar urethroplasty) had to be at least 12 weeks.

### Surgical Procedure

The perioperative management was based on a standardized institutional protocol; each patient received perioperative i.v. antibiotic therapy (cefuroxime and gentamicin). The AUSs were implanted according to standardized approaches by high-volume surgeons.

**Figure 1:**
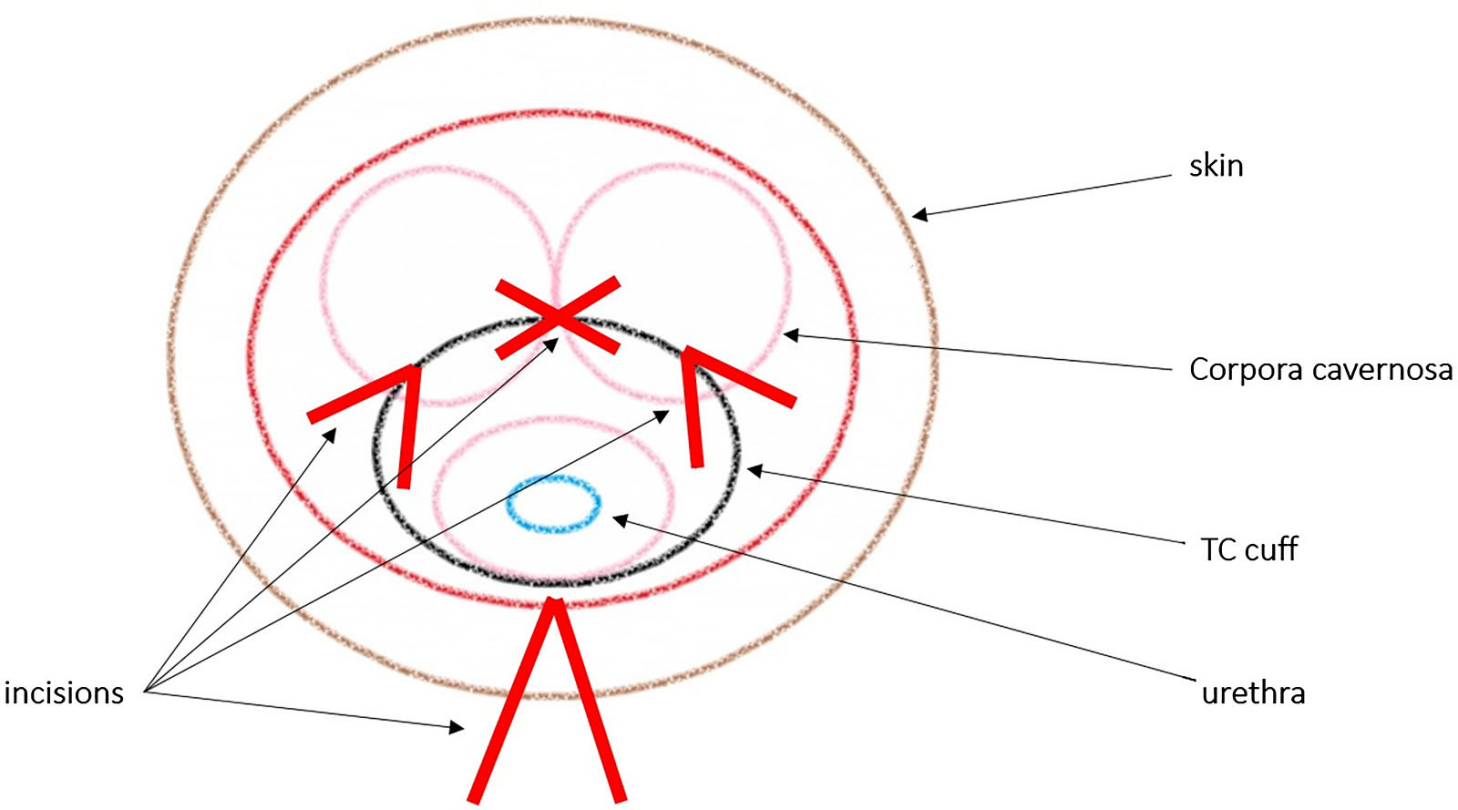
Transversal view of transcorporal (TC) cuff placement.

For the TC implantation, patients were placed in a lithotomy position. A midline perineal incision was performed with a subsequent dissection in order to expose the urethra and adjacent lying corpora cavernosa. The urethra was prepared without excessive mobilization. Cuff placement was 2–3 cm distally to the original cuff location or site of urethroplasty. Two longitudinal incisions were made into the tunica albuginea of both corpora cavernosa, lateral to the urethra. In order to create a tunnel inside the corpora cavernosa, blunt dissection was performed between the two corporal incisions (see Figure 1). Cuff placement was performed after circumference measurements. After cuff placement, the tunica albuginea was sutured. The pump and the balloon were implanted in the scrotum and abdomen. The AMS 800 system was deactivated after the procedure, and a 12-F transurethral catheter was placed and left in situ for 3 days after surgery. Postvoid residual urine measurements after catheter removal and radiological baseline studies were performed. The AUS activation was performed 6 weeks after implantation.

### Follow-Up

FU was performed according to our institutional protocol. All patients were assessed for postoperative hematoma on days 1–5 after surgery and when they were readmitted to our hospital 6 weeks after AUS placement. After radiological imaging control of the sphincter device position, the AMS 800 was activated in an inpatient setting, and the patients were trained to apply the scrotal pump. Functional outcome was objectified by the stress pad test (11), uroflowmetry, postvoid urine measurement, and clinical examination. Furthermore, a standardized, nonvalidated questionnaire was administered, and erectile function was evaluated. For FU, patients were advised to return to our hospital at 6 and 24 months after surgery and thereafter 2 two years.

### Study Endpoint

The primary endpoint was the assessment of TC explantation rates. The explantation-free survival was defined as patients without any need for explantation of the AMS 800 system during FU.

The secondary endpoint of the study was the continence rate after AMS 800 implantation. The level of SUI was assessed by the 1-h stress pad test (urine loss in g) and the number of pads used per day. Objective or social continence was defined as the use of 0 pads/day or <2 pads/day, respectively.

### Statistical Analyses

The probability of explantation-free survival was calculated using the Kaplan–Meier curve. Statistical tests were performed with SPSS 20 (SPSS Inc., IBM Corp., Armonk, NY, USA) and R version 3.5.1 (The R Foundation).

## Results

### Patient Characteristics

Patient characteristics are summarized in Table 1. Overall, 39 patients were analyzed. The median age at surgery was 73 years. Overall, radical prostatectomy, TUR-prostate, and radical cystectomy were performed in 84.6%, 10.3%, and 2.6% of the cases, respectively. One patient had a history of pelvic trauma. About 61.5% of the patients had a history of pelvic radiation. The majority of patients had more than one previous urethral surgery (46.2%, 2; 12.8%, 3).

**Table 1:** Clinical characteristics in TC patients treated by AUS implantation.

Patients, *n* (%)		*n* = 39 (100.0)
Median age at surgery, years (IQR)		73.0 (68–76)
Comorbidities/previous surgeries, *n* (%)		
	Diabetes mellitus	6 (15.4)
	No. of previous surgery (IQR)	2 (1–2)
Surgeries prior SUI, *n* (%)		
	Radical prostatectomy	33 (84.6)
	TURP	4 (10.3)
	Radical cystoprostatectomy	1 (2.6)
	Trauma	1 (2.6)
Pelvic radiation therapy, *n* (%)		24 (61.5)
Surgeries prior artificial urinary sphincter implantation, *n* (%)		
	Open surgical therapy for SUI	37 (94.8)

*AUS, artificial urinary sphincter; IQR, interquartile range; SUI, stress urinary incontinence; TC, transcorporal cuff; TURP, transurethral resection of the prostate*.

The median cuff size applied was 4.5 cm.

### Functional Outcome and Explantation Rate

The subjective, objective, and social continence rates after 27 months of median FU [24–41 m] were 69.7%, 54.5%, and 78.8%, respectively (Table 2). Median pad usage was 1 pad/day [1–2.5].

**Table 2:** Explantation rate, continence, and erectile function.

Patients, *n* (%) (Intention to treat)	*n* = 39 (100.0)
Explantation (%)	9 (23.1)
Objective continence (%)	18 (46.2)
Subjective continence (%)	23 (59.0)
Social continence (%)	26 (66.7)
Erectile function before surgery (%)	6 (15.4)
Erectile function after surgery (%)	3 (7.7)

Preoperatively, sufficient erectile function for sexual intercourse or masturbation was present in 15.4% of the patients, compared to 7.7% after TC placement according to the aforementioned technique.

Only 2.6% of the patients had a relevant perineal hematoma after TC placement.

**Figure 2:**
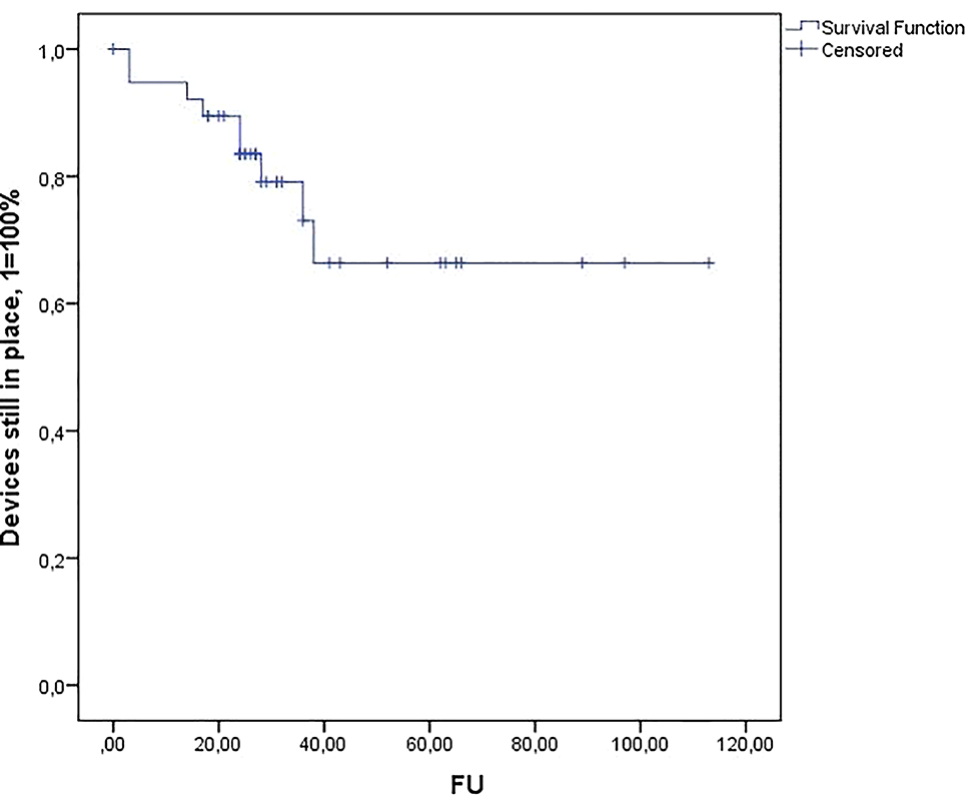
Kaplan–Meier estimates of explantation-free survival after TC artificial urinary sphincter implantation.

According to the Kaplan–Meier analysis (Figure 2), 5 years after implantation, more than 60% of the TC devices were still in place.

In the Kaplan–Meier estimate, the calculated mean durability of the AMS 800 system was 83.4 months [67–100 m, CI 95%]. The median explantation-free time in the TC cohort was 40 months.

## Discussion

The literature on the outcomes of AUS surgery in salvage high-risk cases is scarce.

Studies have shown that reimplant cases are at a higher risk of complications and explantation (7, 8, 12). AUS reimplant cases have a fourfold higher risk for cuff erosion and explantation compared to primary cases (7). History of device explantation is significantly associated with a shorter explantation-free survival (12). Moreover, reimplant cases are associated with worse functional outcomes. It argued that reimplant cases based on TC placement are associated with more infections because of postoperative hematoma as well as a loss of erectile rigidity due to the dissection of the corpora. Thereupon, continence levels are reported to be worse than after primary AUS placement. In order to maximize the longevity of AUS treatment in patients with severe SUI, AUS treatment algorithms should be employed, which keep TC AUS placement as the ultimate option (6, 13).

These aspects bring up the question of whether the TC AUS placement with intraoperative closure of the corpora cavernosa is a viable ultima salvage technique in high-risk cases that allows to maximize explantation-free survival while maintaining erectile rigidity.

The TC technique offers significant advantages in salvage AUS surgery as it protects the compromised urethra from further microcirculation damage by keeping corporal tissue as a sublayer under the cuffs. This is supported by data by Guralnick et al., which shows low explantation rates in the FU [no explantations at 17 months]. Furthermore, studies have also shown convincing functional outcomes with respect to continence for TC placement in revision cases [76%–84% reporting 0–1 pads/day] (14, 15). Magera and Elliott also showed a significant improvement of continence in 69% of the salvage TC cases after a median FU of 26 months (16). Regarding erectile rigidity, the literature is scarce, as many AUS patients suffer from erectile dysfunction due to RRP or radiation therapy long before implantation takes place. It is argued that the TC approach causes erectile dysfunction due to the necessary injury of the corpora cavernosa. However, Wiedemann et al. showed in a study with 23 patients that erectile function could be maintained despite dissection of the corporal body. Four of six patients who had good preoperative erectile function had no deterioration of their IIEF-5 score (15). This is supported by Brant, who argues that closure of the corporal body may prevent postoperative bleedings as well as a loss of erectile rigidity (10).

Our results show that the TC AUS implantation with the closure of the corporal body is a viable salvage method in high-risk patients suffering from severe SUI.

An explantation rate of less than 20% at a median FU of 27 months in a high-risk cohort with patients with a history of up to seven genito-urethral surgeries is within the range of rates published in the literature thus far (13, 17, 18). Only one patient suffered from a relevant postoperative hematoma. With respect to continence, the objective (0 pads/day), subjective, and social (<2 pads/day) continence rates of 69.7%, 54.5%, and 78.8%, respectively, were also within the rates reported in the literature (objective continence 47%–72%; social continence 76%–84%) (14, 15, 19, 20). Overall, the continence results in this study cohort are worse than in the DC AUS study cohorts published before, as the TC AUS is employed as the ultimate salvage option in our department in patients with a history of up to seven genitourethral surgeries (objective continence DC AUS 88%; social continence 94%) (6).

With respect to erectile function, the study results show that, despite the higher degree of invasiveness associated with the TC approach, about 50% of the patients who did not suffer from erectile dysfunction preoperatively did not suffer from a loss of erectile rigidity after TC placement.

In clinical practice, patients ought to be informed about the fact that explantation rates are significantly higher than after primary AUS implantation, which is due to the fact that it is an ultimate salvage approach in our department. However, in view of the good social continence rates and limited alternative treatment options (i.e., perineal closure and suprapubic cystostomy), patients also have to be informed about the opportunity of further viable incontinence treatment.

Strong points of this study are the strict surgical standardization and the standardization of the perioperative management.

However, when analyzing the descriptive data, it ought to be taken into consideration that the TC cohort is small (*n* = 39) and 11 patients were lost to FU. Moreover, in order to further analyze the functional impact of the closure of the corporal bodies in more detail, a prospective and randomized trial would be necessary. Further studies should also address the lack of a control group (i.e., closure of the corporal body vs. nonclosure of the corporal body), which constitutes a central limitation of this paper.

## Conclusions

The TC approach with a closure of the corporal bodies is a viable salvage option for high-risk AUS patients. It constitutes an option with considerably low explanation rates and good functional outcomes with respect to social continence. Erectile function can be maintained in about 50% of the cases. Nevertheless, the TC approach should be regarded as the ultimate salvage approach due to its invasiveness and worse continence rates than in a distal DC setting.

## Data Availability

The original contributions presented in the study are included in the article/supplementary material; further inquiries can be directed to the corresponding author/s.
